# An Unexpected Presentation of Serotonin Syndrome in a Patient Receiving ECT

**DOI:** 10.1155/2024/6938553

**Published:** 2024-08-23

**Authors:** Shahzaib Khan, Breanna Wennberg, Malgorzata Witkowska, Jacob Rattin, Raunak Khisty

**Affiliations:** ^1^ Riverside Medical Center, 350 N Wall St, Kankakee 60901, IL, USA; ^2^ Cleveland Clinic Foundation, Cleveland, USA

## Abstract

Serotonin syndrome is a toxidrome consisting of autonomic instability, altered mentation, hyperreflexia, clonus, and seizures. It is suspected to be due to either elevated serotonin concentrations or overstimulation of 5-hydroxytryptamine (5-HT) receptors. There are at least seven families of serotonin or 5-HT receptors along with multiple subtypes. The 5-HT_1A_ and 5-HT_2A_ serotonin receptor subtypes are heavily suspected to cause the broad spectrum of symptoms seen in serotonin syndrome. We present the case of a young woman treated with multiple psychotropic medications who developed serotonin syndrome (SS) after receiving electroconvulsive therapy (ECT). She had multiple psychiatric hospitalizations, and ECT was determined to be the appropriate course of treatment due to her treatment-resistant symptoms and catatonia. The case was unique as she tolerated multiple ECT treatments over a few weeks before the acute onset of serotonin syndrome following her eighth treatment, and she did not have any medication changes after the second ECT treatment. The patient's acute presentation of rigidity, elevated temperature, hyperreflexia, diaphoresis, confusion, and psychomotor agitation led to a diagnosis of serotonin syndrome. ECT is a neuromodulatory procedure approved for treatment-resistant depression and schizophrenia that involves electrically stimulating the brain with electrodes on the scalp to induce a seizure. The mechanism by which ECT confers therapeutic benefit for patients with neuropsychiatric conditions is not entirely understood. We discuss some of the literature on SS and ECT to better understand the potential for a causal relationship.

## 1. Introduction

Serotonin syndrome (SS) is a toxidrome consisting of autonomic instability, altered mentation, hyperreflexia, clonus, and seizures [1, 2]. It is suspected to be due to either elevated serotonin concentrations or overstimulation of serotonin receptors as serotonin is involved in various physiologic functions including thermoregulation, platelet aggregation, bronchoconstriction, vasoconstriction, gastrointestinal motility, attention and cognition, behavior, memory, uterine contraction, migraines, sexual behavior, and pain [1, 3]. There are at least seven families of serotonin or 5-hydroxytryptamine (5-HT) receptors, some of which have multiple subtypes, resulting in a total of 14 structurally and pharmacologically distinct receptors [3]. Of these, overactivation of the 5-HT_1A_ and 5-HT_2A_ subtypes is more implicated in developing SS [3, 4, 5]. 5-HT_2A_ is thought to be involved in hypertonicity and hyperthermia, while 5-HT_1A_ may contribute to milder symptoms of anxiety and hyperactivity [3]. Most antidepressant medications either increase synaptic serotonin concentrations or stimulate postsynaptic 5-HT receptors, thereby placing patients at an increased risk of developing SS. Many anxiolytics and antipsychotics also work via the action of serotonin receptors [5]. Several nonpharmacological factors, including nonprescribed drug use and specific dietary and supplement intake, can interact with medications and individual metabolic and genetic variations to influence serotonin levels in the nervous system [3, 6].

Electroconvulsive therapy (ECT) is a neuromodulatory procedure approved for treatment-resistant depression and schizophrenia. It involves electrically stimulating the brain with electrodes on the scalp to induce a brief, controlled seizure.

We describe the case of a patient who developed SS acutely after receiving ECT. She had a history of anxiety and depression and was on multiple serotonergic antidepressant medications while on maintenance ECT for refractory symptoms. She had a long-standing history of being on psychiatric medications and had successfully completed multiple sessions of ECT prior to developing SS. This made the onset of her symptoms unexpected. Hypersensitivity to psychotropic medications during a course of ECT is thought to be rare, and the pathophysiology is poorly understood. Our case demonstrates that even patients that have previously had positive therapeutic benefit and minimal adverse effects with ECT are at risk of developing SS.

## 2. Case Presentation

A 34-year-old woman with a past psychiatric history of major depressive disorder (MDD), generalized anxiety disorder (GAD), panic disorder, bulimia nervosa, posttraumatic stress disorder (PTSD), self-harm, and prior suicide attempts was brought to the emergency department for an acute panic attack. She was unable to respond to any questions and was unable to follow commands. Her vitals were stable, and she was diaphoretic, did not display tracking eye movements, was rocking back and forth, and displayed verbigeration by repeatedly saying “yes.” She appeared to be responding to internal stimuli. She had visible bruises on her left upper extremity. She had similar prior episodes and historically improved with lorazepam. Her presentation was consistent with catatonia, and she had a score of 13 on the Bush–Francis Catatonia Rating Scale (BFCRS). The BFCRS is a 23-item rating scale and screening tool to assist with diagnosing catatonia; a score of two or more on the screening portion is considered a positive diagnosis [7].

She was known to have poor coping and stress management skills and had three psychiatric hospitalizations within the past year. She followed regularly with her outpatient psychiatrist, was compliant with medications, and was involved in regular individual therapy. Prior medication trials included duloxetine, bupropion, prazosin, ropinirole, clonazepam, quetiapine, lurasidone, mirtazapine, trazodone, fluoxetine, hydroxyzine, venlafaxine, and paroxetine. She had adverse reactions to haloperidol, paroxetine, and venlafaxine. Her current medications included buspirone 20 mg three times daily, duloxetine 90 mg daily, lamotrigine 25 mg daily, and clonazepam 0.5 mg twice daily. A urine drug screen was positive only for benzodiazepines which was expected since she was prescribed clonazepam.

Prior to initiation of ECT, buspirone was increased to 30 mg three times daily. Clonazepam was increased to 0.5 mg three times daily, and she was started on ECT during the hospitalization. ECT was recommended due to poorly controlled mood symptoms and repeated catatonic episodes. Lamotrigine, which was a historic medication started by her outpatient provider for adjunct treatment of depression and mood stabilization due to multiple medication failures, was discontinued to limit impact on seizure quality during treatment. She received ECT three times weekly throughout the course of her treatment. Prior to treatment, the patient was premedicated with flumazenil to limit any potential effect on seizure quality. Duloxetine was increased to 120 mg daily to optimize treatment after the second session of ECT. Her anxiety and symptoms improved gradually. Her medication regimen remained duloxetine, buspirone, and clonazepam without further changes. She received three ECT treatment sessions while hospitalized which she tolerated well. She initially had significant nausea and vomiting after the first session of ECT but otherwise tolerated the procedure and medication changes.

After receiving three sessions of ECT while inpatient, she continued treatment on an outpatient basis. Two weeks after hospital discharge, the patient underwent her eighth treatment session of ECT (see Supplementary Materials for details of ECT parameters and response during this treatment session). Approximately 1 hr after ECT treatment, the patient alarmed nursing staff as she was not following commands and repeatedly saying “yes,” similar to her initial hospital presentation. Her pupils were equal and dilated with reaction to light. She was diaphoretic with a recorded temperature of 100.4 F, corresponding to 38.0 C. She displayed hyperreflexia and psychomotor agitation. She was restlessly moving her lower extremities. She received 1 mg of midazolam intravenously, but there was minimal immediate effect. Differential diagnoses included anticholinergic syndrome and neuroleptic malignant syndrome, but these were both considered unlikely as she had not had any known current exposure to neither anticholinergic nor neuroleptic medications. A head computed tomography showed no acute processes. A diagnosis of SS was made given her symptoms of confusion, acute onset, rigidity, elevated temperature, hyperreflexia, diaphoresis, and psychomotor agitation.

We ordered lorazepam 2 mg intravenously every 4 hr with the first dose given immediately. The patient was transferred to the intensive care unit (ICU) for further observation and supportive care. She was started on intravenous fluids. We considered using cyproheptadine, but it was not needed as the patient responded well to lorazepam and was resting comfortably 2 hr later. She returned to baseline and was discharged from the ICU after 24 hr.

## 3. Discussion

We found multiple case reports describing patients receiving ECT and subsequent development of SS [2, 8, 9, 10, 11]. It is unclear if ECT or other neuromodulatory procedures can contribute to developing SS in the absence of serotonergic medications as many patients are concurrently on antidepressants or other psychiatric medications for maintenance or to prevent worsening of symptoms [2]. The precise incidence of SS is also unknown since it is a clinical diagnosis without confirmatory testing [1, 3]. However, reported incidences and deaths are increasing as serotonin-modifying medications become more clinically used [3]. Antipsychotics, antiemetics, migraine medications, and drugs of abuse like fentanyl and lysergic acid diethylamide all include serotonin receptor activity [3]. SS symptom onset is typically acute within 24 hr of exposure to the offending agent, with uncomplicated cases resolving within 72 hr [3]. The classic triad of symptoms include altered mental status, autonomic overactivity, and neuromuscular hyperactivity, but the severity can range from mild to life-threatening [3]. Symptoms may also be dose dependent; most cases involve the use of monoamine oxidase inhibitor (MAOI) drugs as they confer the greatest risk by causing larger increases in serotonin concentration [3, 5, 12]. Criteria aiding in the diagnosis of SS have been proposed by Sternbach [13], Hegerl et al. [14] (Serotonin Syndrome Scale), Radomski et al. [15], and Dunkley et al. [16] (Hunter Serotonin Toxicity Criteria).

Management is predominantly supportive care with discontinuation of serotonergic medications. Treatment involves intravenous fluids, benzodiazepines, sedation, and muscle paralysis and intubation in severe cases [3]. There is greater suspicion for 5-HT_2A_ to cause hyperthermia as 5-HT_2A_ antagonists can help manage hyperthermia [5, 12]. Cooling measures have been observed to reduce the activity of 5-HT_2A_ receptors and attenuate the response to prevent the most severe symptoms [3, 12]. Benzodiazepines can help relieve the milder symptoms of anxiety and hyperactivity related to 5-HT_1A_ receptor activation [3]. A serotonin antagonist such as cyproheptadine can be considered if supportive interventions are unsatisfactory, but strong evidence to support its use is lacking [1, 3, 4, 17]. There is also anecdotal support for chlorpromazine given its 5-HT_2A_ antagonism [3, 4, 18]. Treatment related to 5-HT_2A_ receptor antagonism is aimed at relieving hyperthermia [3].

The mechanism by which ECT leads to therapeutic benefit is poorly understood, but there are many hypotheses. ECT may enhance 5-HT receptor sensitivity, thereby placing patients on serotonergic medication at risk [2]. It is also suspected that ECT may enhance the permeability of the blood–brain barrier which could lead to drastic increases in drug concentrations shortly after the treatment [2, 11]. As such, a patient receiving ECT while taking serotonergic medications can be at risk for supratherapeutic serotonin levels resulting in SS, which is the suspected etiology in our patient. Severe presentation of SS is a serious and potentially life-threatening condition. Relatively long wash-out periods of some causative medications and a wide array of other factors influencing serotonin levels can contribute to its development. The pursuit of optimal clinical practice demands rigorous attention to both the precise and individualized management of medications. It may be worth considering a temporary medication dose reduction while the patient is undergoing active ECT treatment.

## 4. Conclusion

With the prevalence of complex, multidrug therapies, it is important to be mindful of the possibility of developing SS, especially in patients receiving ECT with the concurrent use of serotonergic agents. This can occur even in the absence of medication changes and even in patients that have tolerated several sessions of ECT previously. Providers should be familiar with the signs and symptoms of SS and how to treat it. Patients should also be made aware of the risk, especially as serotonin-modifying medications become increasingly more used and patients have easier access to supplements that can modify the pharmacokinetics of drug metabolism.

## Data Availability

There is no identifiable data associated with this report.

## Supplementary Materials

Supplementary Materials Details of ECT parameters and response during treatment #8.
