# Supplementary material for: An Unexpected Presentation of Serotonin Syndrome in a Patient Receiving ECT
Source: Case Rep Psychiatry. 2024 Aug 23;2024:6938553. doi: 10.1155/2024/6938553 (PMC11364471; doi:10.1155/2024/6938553)
Supplement: Supplementary Materials — Details of ECT parameters and response during treatment #8. [file 6938553.f1.docx]

**Supplementary material:** Details of ECT parameters and response during treatment #8

Anesthesia Medications: Flumazenil (Romazicon) 0.5 mg/5mL injection, Methohexital (Brevital sodium) 1 mg/kg injection, and Succinylcholine (Anectine) 20 mg/mL injection

Other Medications Given Prior to Procedure: Toradol and Ondansetron

Nasopharyngeal Airway: No

History of Postictal Agitation: No

Electrode Placement: Right unilateral

Frequency: 60 Hz

Pulse Width: 0.3-0.37 ms

Stimulus Duration: 8 sec

Stimulus Current: 800 mAmp

Charge: 230.4 mC

Motoric Seizure Length: 20 sec

EEG Seconds: 34 sec

Quality of Seizure Score: low amplitude, good postictal suppression, fair length

ECT Results: Clinical seizure was satisfactory, Patient tolerated ECT well

Patient Condition Post ECT: Stable

Parameters for the next ECT: Patient being treated at 6X seizure threshold
